# Isolation of Bacterial Plasmid-Related Replication-Associated Circular DNA from a Serum Sample of a Multiple Sclerosis Patient

**DOI:** 10.1128/genomeA.00847-14

**Published:** 2014-08-28

**Authors:** Karin Gunst, Harald zur Hausen, Ethel-Michele de Villiers

**Affiliations:** Division for the Characterization of Tumorviruses, Deutsches Krebsforschungszentrum, Heidelberg, Germany

## Abstract

*Psychrobacter* species are considered to be opportunistic human pathogens. We report here the isolation of a circular DNA molecule, MSSI1.162, from a serum sample taken from a multiple sclerosis patient during relapse. This isolate is distantly related to known *Psychrobacter* species and their plasmids.

## GENOME ANNOUNCEMENT

*Psychrobacter* species are frequently present as food contaminants and have been isolated from human tissues, including the brain, cerebrospinal fluid, and blood. They are considered to be opportunistic human pathogens (1, 2). We report here the isolation of a circular DNA molecule, MSSI1.162, from a serum sample taken from a multiple sclerosis patient during relapse.

Rolling circle amplification and restriction digestion (3) were performed on DNA extracted from serum samples from patients with multiple sclerosis. The resulting fragment was cloned into vector pUC19. The full-length genome was verified by inverted PCR amplification using primers designed on the sequence identified initially: forward primer 5′-GACTTCTGATTGATTGATGCCTG-3′ and reverse primer 5′-CCTGTTGAATACCGCTTAAATACT-3´. All products were sequenced by primer walking. MSSI1.162 (MSSI, multiple sclerosis serum isolate) (1,627 bp) shares 66% nucleotide similarity by BLASTn analysis with the pRWF102 plasmid of *Psychrobacter* sp. PRwf-1. The putative protein (321 amino acids) encoded by the large open reading frame (ORF) shows weak similarity to the replication protein family of *Escherichia coli*, as analyzed by ProtSweep (4), and it has 60% similarity to the Rep protein of *Psychrobacter* sp. PRwf-1 plasmid pRWF102.

This new isolate is only distantly related to known *Psychrobacter* species and their plasmids. It may therefore represent a yet unknown human pathogen.

### Nucleotide sequence accession number.

The complete sequence of MSSI1.162 is available in the EMBL Databank under the accession no. LK931486.
